# Supplementary material for: Mechanically activated ion channel Piezo1 modulates macrophage polarization and stiffness sensing
Source: Nat Commun. 2021 May 31;12:3256. doi: 10.1038/s41467-021-23482-5 (PMC8167181; doi:10.1038/s41467-021-23482-5)
Supplement: Supplementary file 1 — Supplementary Information [file 41467_2021_23482_MOESM1_ESM.pdf]

Supplementary Information for:

Mechanically activated ion channel Piezo1 modulates macrophage polarization and stiffness sensing

Hamza Atcha[1,2], Amit Jairaman[3], Jesse R. Holt[3,4], Vijaykumar S. Meli[1,2,5], Raji R. Nagalla[1,2], Praveen Krishna Veerasubramanian[1,2], Kyle T. Brumm[1,2], Huy E. Lim[1,2], Shivashankar Othy[3], Michael D. Cahalan[3], Medha M. Pathak[1,3,4], and Wendy F. Liu[1,2,5,*]

[1] Department of Biomedical Engineering
[2] The Edwards Lifesciences Center for Advanced Cardiovascular Technology
[3] Department of Physiology and Biophysics
[4] Sue and Bill Gross Stem Cell Research Center
[5] Department of Chemical Engineering and Materials Science

University of California Irvine
2412 Engineering Hall
Irvine, CA 92697
Tel: (949) 824-1682
Fax: (949) 824-9968
Email: wendy.liu@uci.edu

[*] To whom correspondence may be addressed.

Supplementary Tables

Supplementary table 1: List of antibodies with dilutions and vendors used for this study. Abbreviations: WB – Western blot, IF – Immunofluorescence, IHC – immunohistochemistry.

**Antibody**	**Dilution (Application)**	**Vendor (Cat. #)**
iNOS	1:1000 (WB), 1:100 (IHC)	Abcam (ab15323)
ARG1	1:1000 (WB), 1:50 (IHC)	Abcam (ab60176)
GAPDH	1:2000 (WB)	BioLegend (607902)
PIEZO1	1:1000 (WB)	ProteinTech (15939-1-AP)
RFP	1:1000 (WB), 1:400 (IF)	Rockland (600-401-379)
NFκB	1:1000 (WB), 1:800 (IHC)	Cell Signaling (8242S)
p- NFκB	1:1000 (WB)	Cell Signaling (3033S)
NFκB	1:100 (IF)	Santa Cruz Biotech (sc-8008)
STAT6	1:1000 (WB)	Cell Signaling (5397S)
p-STAT6	1:1000 (WB)	Cell Signaling (56554S)
STAT1	1:1000 (WB)	Cell Signaling (14994)
p-STAT1	1:1000 (WB)	Cell Signaling (9167)
STAT3	1:1000 (WB)	Cell Signaling (9139)
p-STAT3	1:1000 (WB)	Cell Signaling (9145)
F4/80	1:200 (IHC)	Fisher Scientific (50-112-9624)

Supplementary table 2: List of primers used for this study.

**Gene Name**	**Forward Primer (5'-3')**	**Reverse Primer (5'-3')**
Piezo1	GTTACCCCCTGGGAACATCT	TTCAGGAGAGAGGTGGCTGT
Piezo2	CTCACCTTTCCTGGCGTCAT	CCTCTTGAAACTCAGGCAGT
Hvcn1	CATCTGACCCAACACCACAG	CAGCTTCTTCTTCCCGTTTG
Trpm7	CCTCATGAAGACCATTTTCTAA	ACAACTGTAACCTTCCTCACAG
Trpv2	TTAAATGACTTGTGAGGGAGATAGC	CAAGTAACACAATCTACCCAAGGTC
Trpv4	TCACCTTCGTGCTCCTGTTG	AGATGTGCTTGCTCTCCTTG
Trpc1	GCCATCTTTGTCACCAGGTT	GCTCGAGCAAACTTCCATTC
Trpc6	GCAGGATTTCGTTGTTGGT	TGCTGACAGTTGGATGAGC
Trek1	ATTGTGCATGGTGACCTCAA	TGCTGACAGTTTGGATGAGC
Il6	CTGCAAGAGACTTCCATCCAGTT	GAAGTAGGGAAGGCCGTGG
Nos2	GAATCTTGGAGCGAGTTGTGG	TTGTACTCTGAGGGCTGACAC
Mcp1	CAGCCAGATGCAGTTAACGC	GCCTACTCATTGGGATCATCTTG
Il1b	GATCCCAAGCAATACCCAAAG	CTTGTGCTCTGCTTGTGAGG
Retnla	GCCAATCCAGCTAACTATCCC	AGTCAACGAGTAAGCACAGG
Mrc1	TGTTTTGGTTGGGACTGACC	TGCAGTAACTGGTGGATTGTC
Arg1	CTCTGTCTTTTAGGGTTACGG	CTCGAGGCTGTCCTTTTGAG
Gapdh	GTCAAGCTCATTTCCTGGTAT	TCTCTTGCTCAGTGTCCTTGC

Supplementary Figures

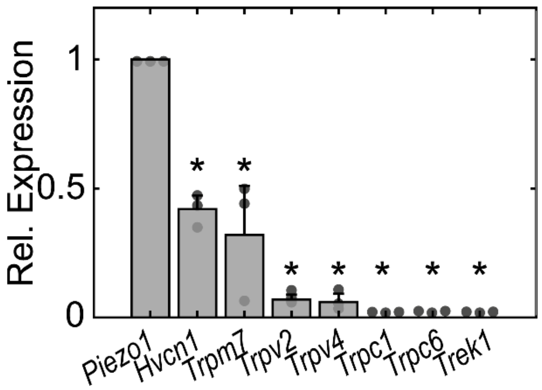

Supplementary Figure 1: Piezo1 is highly expressed in murine BMDMs. Relative gene expression of known mechanosensitive ion channels in BMDMs as measured by qPCR. Gene expression is normalized to the expression of *Piezo1*. Error bars indicate standard deviation for three independent experiments and * $p < 0.05$ when compared only to *Piezo1* expression as determined by a two-tailed paired t-test with Bonferroni correction used for multiple comparisons. Source data including exact p-values are provided as a Source Data file.

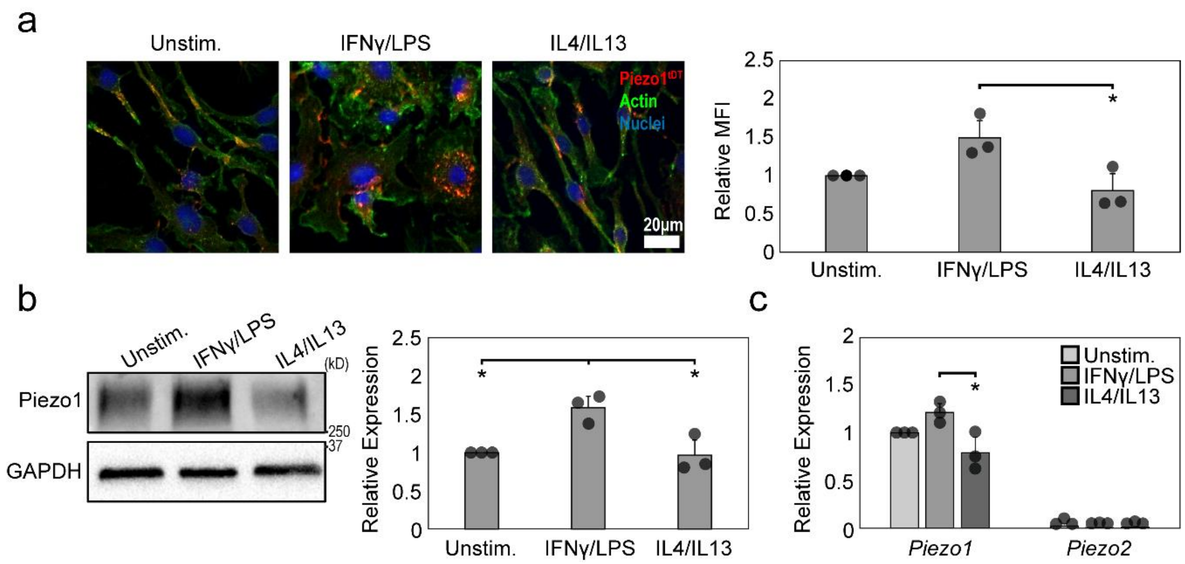

Supplementary Figure 2: Macrophage activation influences Piezo1 expression. (a) Representative immunofluorescence images (left) and quantification of relative PIEZO1 mean fluorescence intensity (MFI) of BMDMs isolated from Piezo1$^{P1\text{-}tdT}$ mice and incubated with media (Unstim.), 0.3ng/mL IFNγ/LPS, or 0.1ng/mL IL4/IL13. MFI is normalized to Unstim. condition. Error bars indicate standard deviation for three independent experiments and * $p < 0.05$ when compared to the corresponding condition by a two-tailed Student's t-test. (b) Representative Western blots (left) and quantification (right) of *Piezo1$^{P1\text{-}tdT}$* expression. Data normalized to Unstim. condition. Blots were obtained from loading equal amounts of protein in separate gels (4% and 4-15% polyacrylamide gels for PIEZO1 and GAPDH blots, respectively) and the resulting blots were processed in parallel. Error bars indicate standard deviation for three independent experiments and * $p < 0.05$ when compared to the corresponding condition by a two-tailed paired t-test. (c) Relative gene expression of *Piezo1 and Piezo2* in Unstim., IFNγ/LPS, and IL4/IL13 stimulated BMDMs. Gene expression is made relative to *Gapdh* and normalized to the expression of *Piezo1* in Unstim. macrophages. Error bars indicate standard deviation for three independent experiments and * $p < 0.05$ when compared to the corresponding condition by a two-tailed Student's t-test. Source data including exact p-values are provided as a Source Data file.

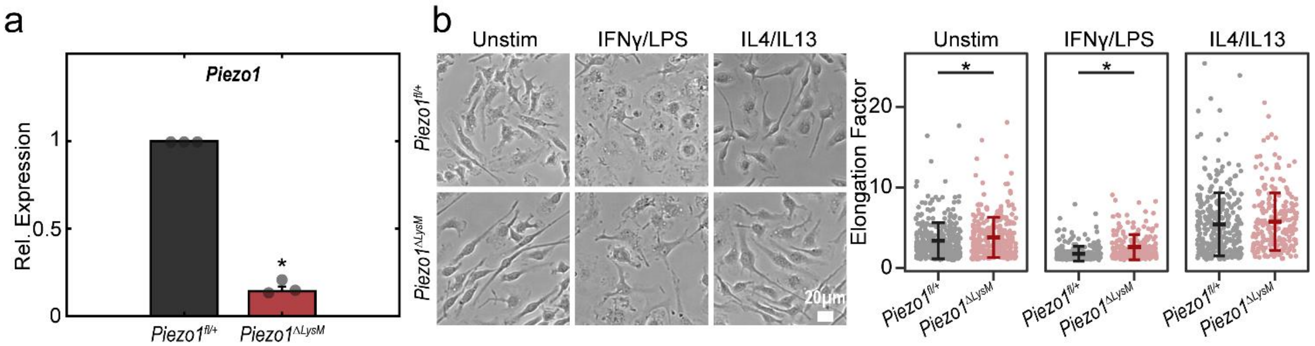

Supplementary Figure 3: Validation of Piezo1 knockout in BMDMs. (a) Relative gene expression of *Piezo1* in unstimulated BMDMs isolated from *Piezo1*$^{fl/+}$ and *Piezo1*$^{\Delta LysM}$ mice, n = 3. Gene expression is normalized to the expression of Piezo1 in BMDMs isolated from *Piezo1*$^{fl/+}$ mice. (b) Representative phase contrast images (left) and quantification of cellular elongation (right) of *Piezo1*$^{fl/+}$ and *Piezo1*$^{\Delta LysM}$ BMDMs treated with media (Unstim.), IFNγ/LPS, and IL4/IL13. N = 363, 306 cells for Unstim., n = 290, 209 cells for IFNγ/LPS, and n = 307, 231 cells for IL4/IL13 *Piezo1*$^{fl/+}$ and *Piezo1*$^{\Delta LysM}$ BMDMs, respectively. Error bars indicate standard deviation for BMDMs harvested from a minimum of three *Piezo1*$^{fl/+}$ and *Piezo1*$^{\Delta LysM}$ mice and * $p < 0.05$ when compared to the *Piezo1*$^{fl/+}$ condition by a two-tailed paired t-test. Source data including exact p-values are provided as a Source Data file.

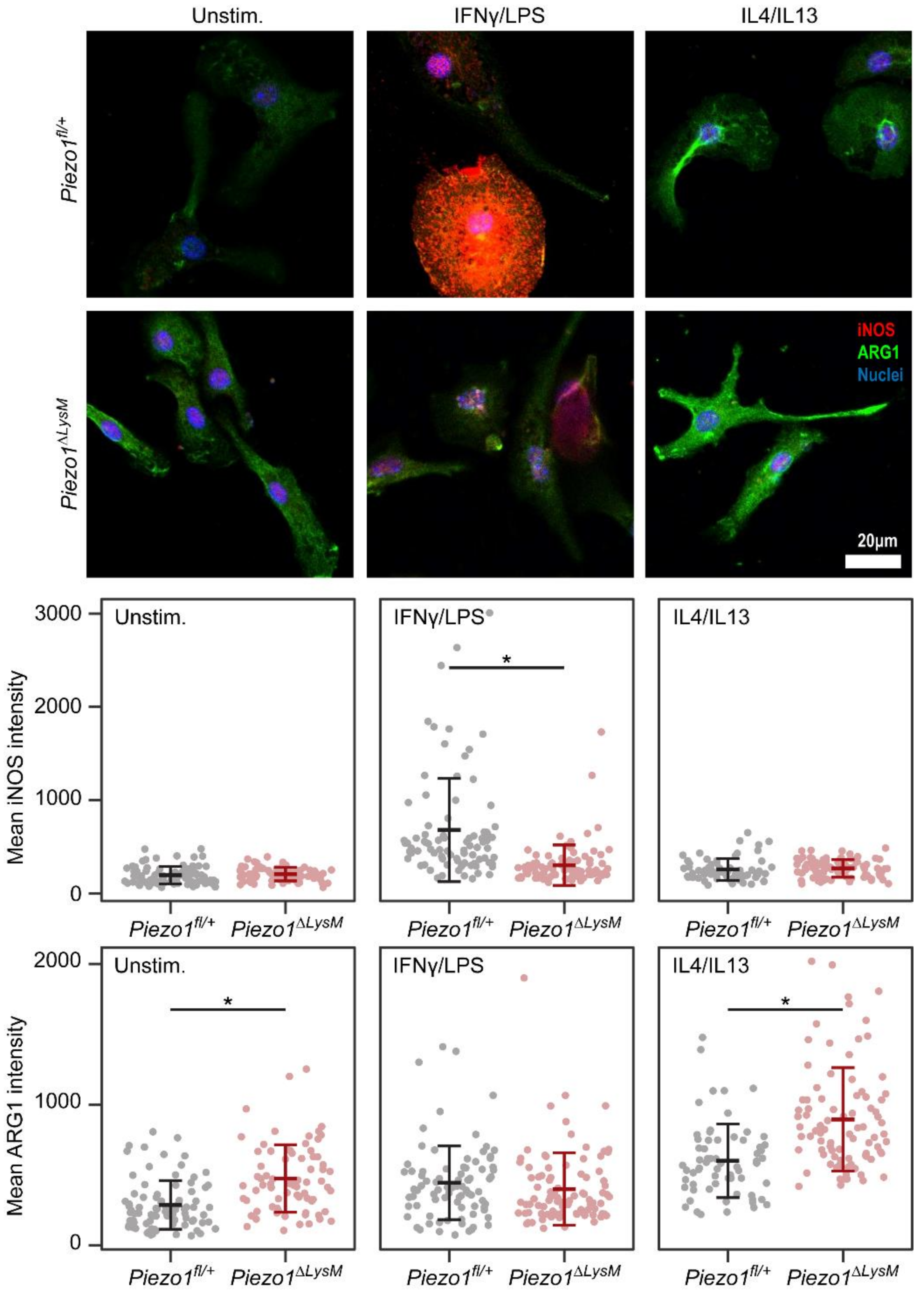

Supplementary Figure 4: Piezo1 deletion suppresses inflammatory and enhances healing activation in peritoneal macrophages. Representative images (top) and quantification of mean iNOS and ARG1 intensity in *Piezo1*$^{fl/+}$ and *Piezo1*$^{\Delta LysM}$ peritoneal macrophages stimulated with media (Unstim.), IFNγ/LPS, and IL4/IL13. N= 85, 75 cells for Unstim., n = 86, 92 cells for IFNγ/LPS, and n = 65, 87 cells for IL4/IL13 treated *Piezo1*$^{fl/+}$ and *Piezo1*$^{\Delta LysM}$ peritoneal macrophages, respectively. Error bars indicate standard deviation for BMDMs harvested from two mice and * $p < 0.05$ when compared to indicated condition by two-tailed Mann-Whitney U test. Source data including exact p-values are provided as a Source Data file.

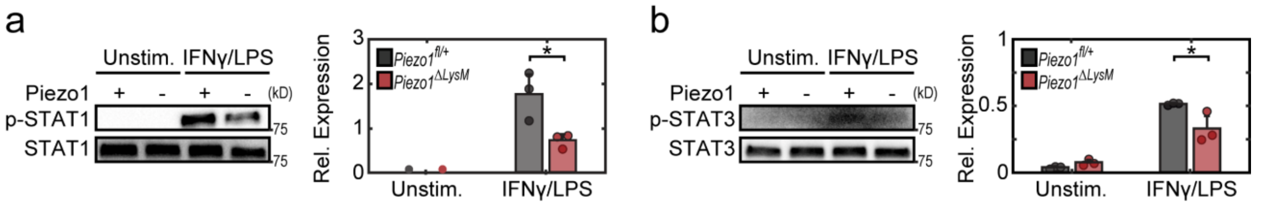

Supplementary Figure 5: Piezo1 regulates activation of STAT1 and STAT3. (a) Representative Western blots (top) and quantification (bottom) of p-STAT1/STAT1 of *Piezo1*$^{fl/+}$ and *Piezo1*$^{\Delta LysM}$ BMDMs incubated with media (Unstim.) or IFNγ/LPS for one hour. (b) Representative Western blots (top) and quantification (bottom) of p-STAT3/STAT3 of *Piezo1*$^{fl/+}$ and *Piezo1*$^{\Delta LysM}$ BMDMs incubated with media (Unstim.) or IFNγ/LPS for one hour. Error bars indicate standard deviation for BMDMs harvested from n = 3 and * $p < 0.05$ when compared to indicated condition by a two-tailed Student's t-test. Phosphorylated and total forms of transcription factors were obtained from loading equal amounts of protein in separate gels and the resulting blots were processed in parallel. Source data including exact p-values are provided as a Source Data file.

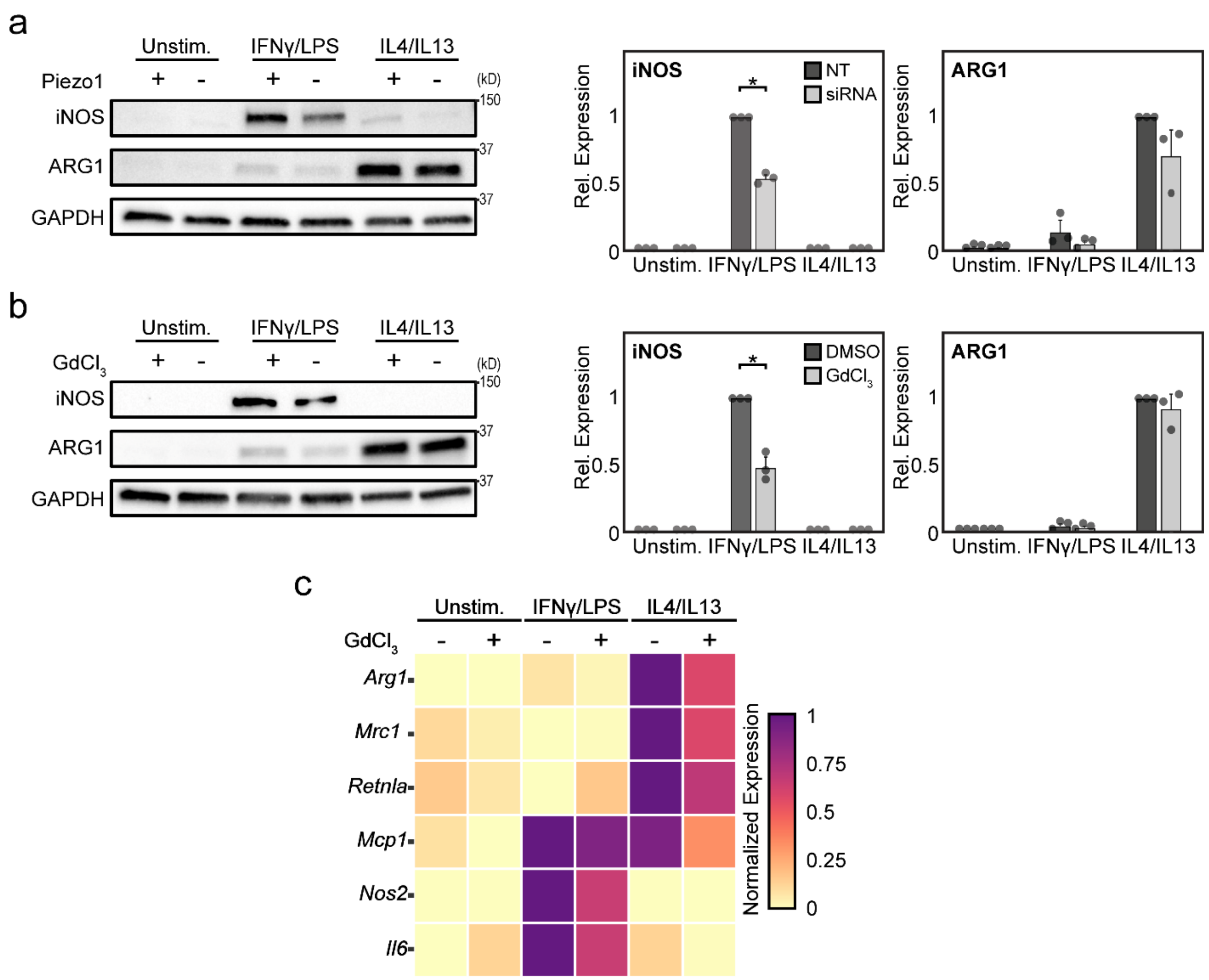

Supplementary Figure 6: Piezo1 inhibition regulates macrophage activation. (a) Representative Western blots (left) and quantification (right) of iNOS, ARG1, and GAPDH of BMDMs treated with control non-target (NT) or Piezo1 siRNA (siRNA) and incubated with media (Unstim.), 0.3ng/mL IFNγ/LPS, or 0.1ng/mL IL4/IL13. (b) Representative Western blots (left) and quantification (right) of iNOS, Arg1, and GAPDH of BMDMs treated with DMSO or 25μM $GdCl_3$ and incubated with media (Unstim.), 0.3ng/mL IFNγ/LPS, or 0.1ng/mL IL4/IL13. (c) Relative gene expression of inflammatory and healing markers in DMSO and $GdCl_3$ treated BMDMs incubated with the indicated conditions, as measured by qPCR. Gene expression is shown relative to the highest expressing condition. Error bars indicate standard deviation for three independent experiments and * $p < 0.05$ when compared to the corresponding condition by a paired t-test. Source data including exact p-values are provided as a Source Data file.

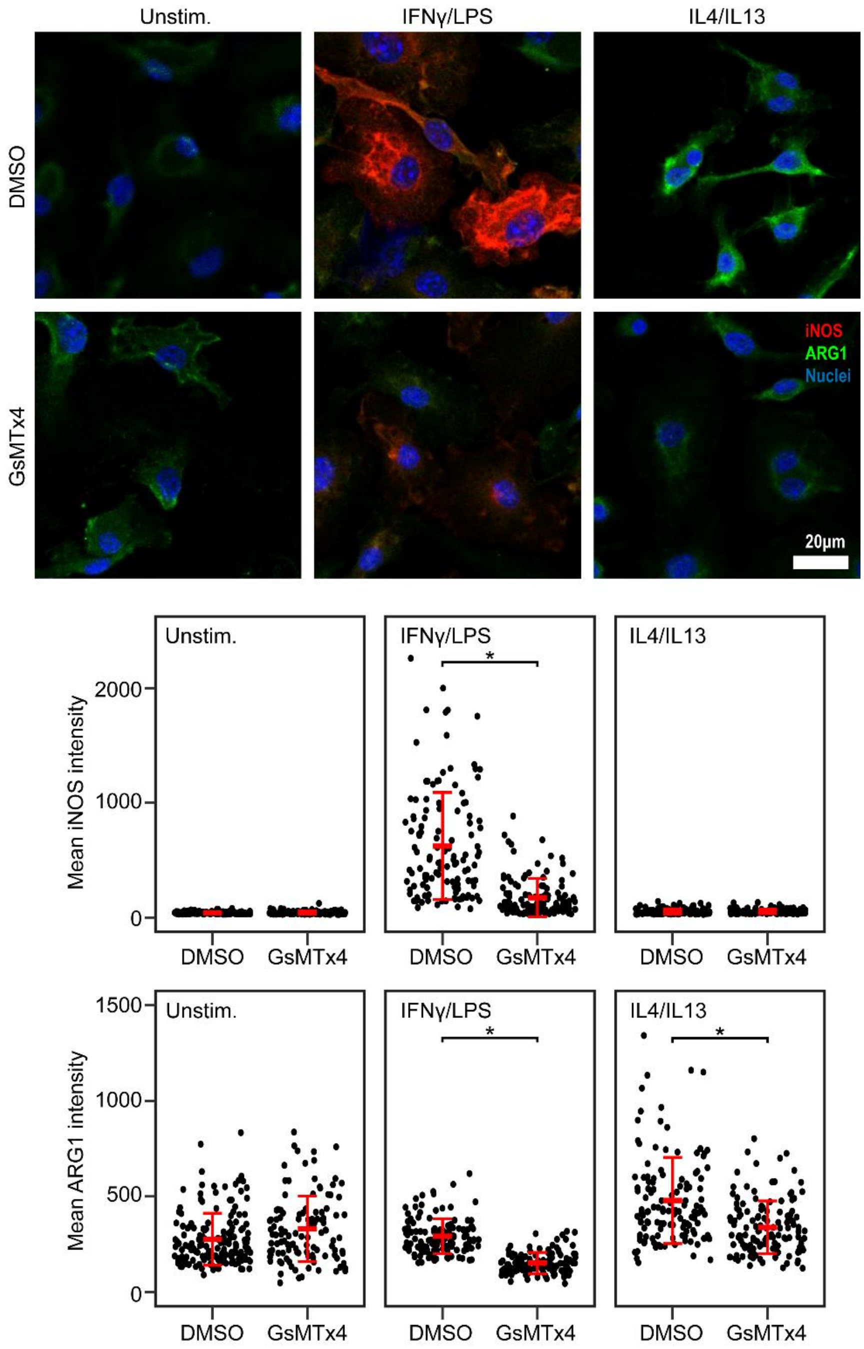

Supplementary Figure 7: GsMTx4 treatment suppresses macrophage inflammatory activation. Representative images (top) and quantification of mean iNOS and ARG1 intensity in wild-type BMDMs treated with 100µM GsMTx-4 in media and stimulated with media (Unstim.), IFNγ/LPS, and IL4/IL13. N= 150-200 cells, error bars indicate standard deviation for BMDMs harvested from three mice and * $p < 0.05$ when compared to indicated condition by two-tailed Mann-Whitney U test. Source data including exact p-values are provided as a Source Data file.

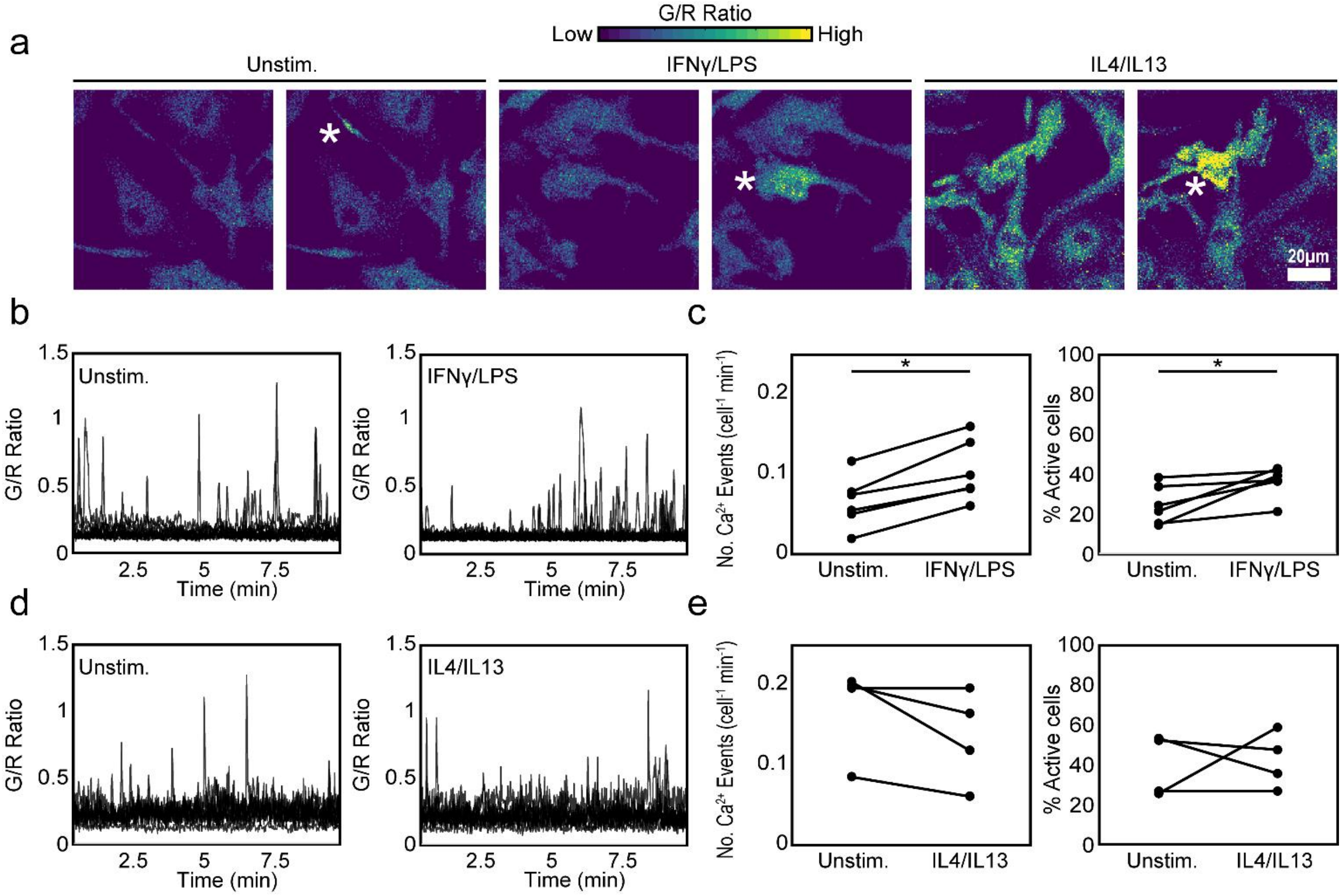

Supplementary Figure 8: Acute addition of IFNγ/LPS but not IL4/IL13 promotes Ca^{2+} activity in Salsa6f+ BMDMs. (a) Representative G/R ratio images taken from a time-lapse video of unstimulated (Unstim.), IFNγ/LPS, or IL4/IL13 stimulated Salsa6f+ BMDMs. Asterisks denote the occurrence of a Ca^{2+} event. (b) Representative Ca^{2+} traces following acute addition of Ringer solution (Unstim.) or Ringer solution containing 100ng/mL IFNγ/LPS in Salsa6f+ BMDMs. (c) Quantification of number of Ca^{2+} events (normalized for cell number and time) and fraction of cells showing Ca^{2+} elevations in BMDMs following acute addition of Ringer solution (Unstim.) or Ringer solution containing 100ng/mL IFNγ/LPS, captured by confocal microscopy. Each paired data point is calculated from a 10-minute time-lapse video (N = 6 videos each) and * $p < 0.05$ as determined by a paired t-test. (d) Representative Ca^{2+} traces following acute addition of Ringer solution (Unstim.) or Ringer solution containing 10ng/mL IL4/IL13 in Salsa6f+ BMDMs. (e) Quantification of number of Ca^{2+} events (normalized for cell number and time) and fraction of cells showing Ca^{2+} elevations in BMDMs following acute addition of Ringer solution (Unstim.) or Ringer solution containing 10ng/mL IL4/IL13, captured by confocal microscopy. Each paired data point is calculated from a 10-minute time-lapse video (N = 4 videos each) and * $p < 0.05$ as determined by a two-tailed paired Student's t-test. Source data including exact p-values are provided as a Source Data file.

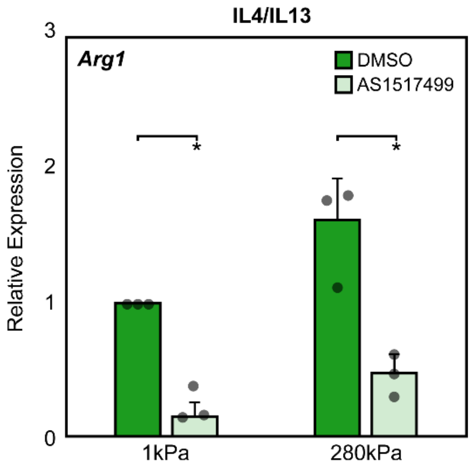

Supplementary Figure 9: Pharmacological inhibition of STAT6 reduces healing activation in IL4/IL13 stimulated BMDMS. Relative *Arg1* gene expression in wild-type BMDMs seeded on 1 kPa and 280 kPa surfaces, exposed to DMSO or 300 nM AS1517499 to inhibit STAT6, and stimulated with IL4/IL13. Data normalized to *Gapdh* and expressed relative to 1kPa DMSO control. Error bars denote Mean ± SD for three separate experiments, * $p < 0.05$ as determined by a two-tailed paired t-test. Source data including exact p-values are provided as a Source Data file.

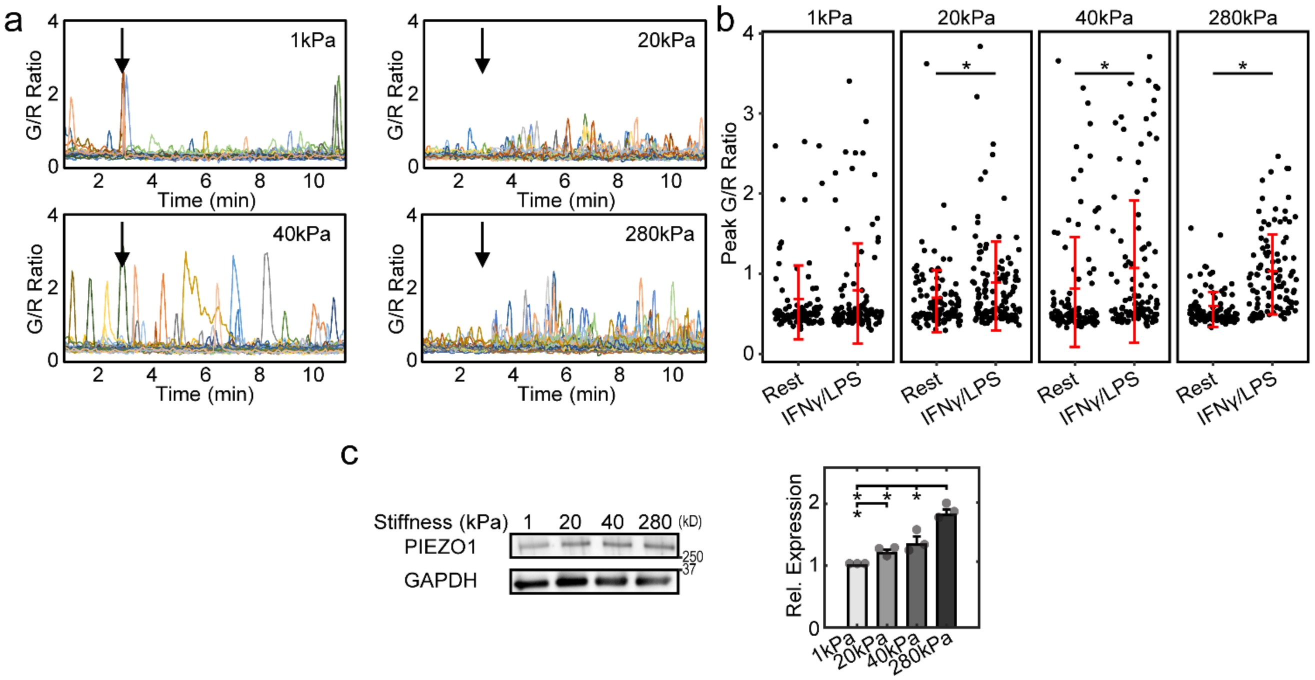

Supplementary Figure 10: Substrate rigidity promotes Ca^{2+} activity and Piezo1 expression. (a) Traces of individual Ca^{2+} events in BMDMs seeded on polyacrylamide gels of 1, 20, 40, and 280kPa stiffness and exposed to Ringer solutions containing 100ng/mL IFNγ/LPS. Arrows indicate addition of cytokines. (b) Quantification of peak G/R intensities of BMDMs on polyacrylamide gels at rest and stimulated with 100ng/mL IFNγ/LPS. N = 102, 118, 113, and 106 for 1, 20, 40, and 280kPa, respectively. Error bars denote standard deviation and * $p < 0.05$ when compared to the corresponding condition as determined by two-tailed Mann-Whitney U test. (c) Representative Western blots (left) and quantification (right) of Piezo1 expression in wild-type BMDMs seeded on 1, 20, 40, and 280kPa polyacrylamide gels. Blots were obtained from loading equal amounts of protein in separate gels (4% and 4-15% polyacrylamide gels for PIEZO1 and GAPDH blots, respectively) and the resulting blots were processed in parallel. Error bars indicate standard deviation for BMDMs harvested from three mice and * $p < 0.05$ when compared to indicated condition by a paired t-test with Bonferroni correction for multiple comparisons. Source data including exact p-values are provided as a Source Data file.

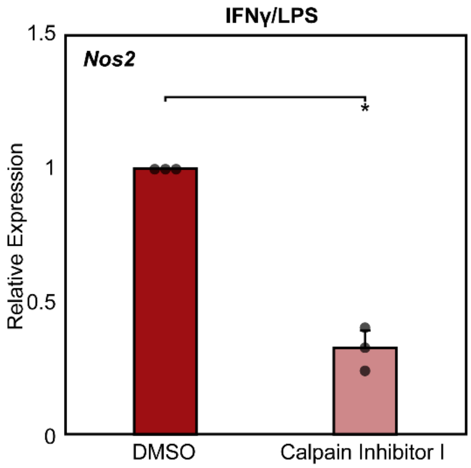

Supplementary Figure 11: Calpains are involved in IFNγ/LPS-mediated inflammation. Relative *Nos2* gene expression in wild-type BMDMs exposed to DMSO or 5µM Calpain Inhibitor I and stimulated with IFNγ/LPS. Data normalized to *Gapdh* and expressed relative to DMSO control. Error bars denote Mean ± SD for three separate experiments, * $p < 0.05$ as determined by a two-tailed paired t-test. Source data including exact p-values are provided as a Source Data file.

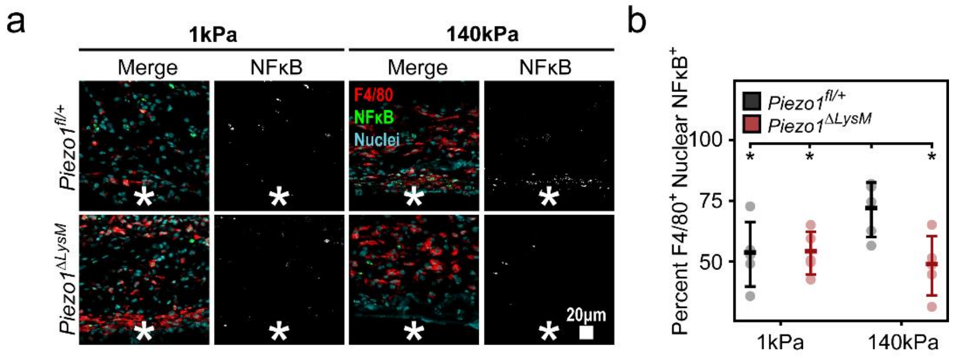

Supplementary Figure 12: Stiffness-mediated Piezo1 activation regulates NFκB translocation *in vivo*. (a) Representative immunohistochemistry images of tissue surrounding soft (1kDa) and stiff (140kDa) PEGDA material implanted in *Piezo1*$^{fl/+}$ and *Piezo1*$^{\Delta LysM}$ mice and stained for F4/80, NFκB, and Hoechst (left). Material location indicated with asterisk. (b) Quantification of percent cells that stained positive for both F4/80 and nuclear localization of NFκB (right) for n = 5. Error bars denote Mean ± SD, * $p < 0.05$ as determined by a two-tailed Student's t-test. Source data including exact p-values are provided as a Source Data file.

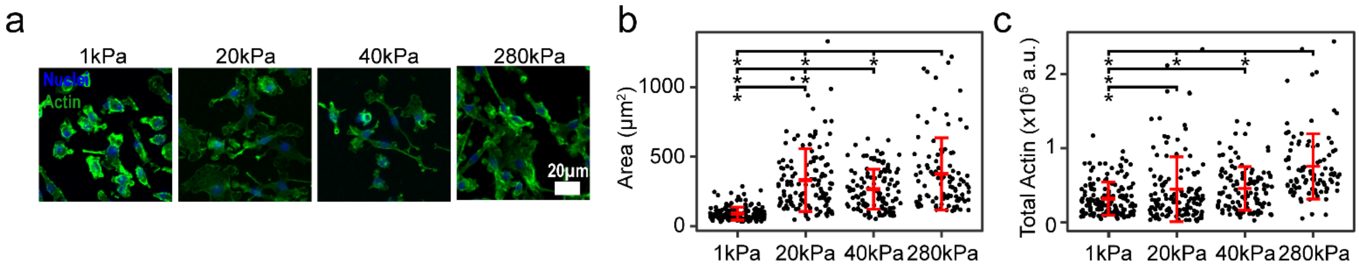

Supplementary Figure 13: Substrate rigidity promotes cell spreading and actin formation. (a-c) Representative images (a) and quantification of area (b) or total actin integrated across cell area (c) in wild-type BMDMs seeded on 1, 20, 40, 280kPa fibronectin conjugated polyacrylamide gels. N = 157, 128, 116, and 121 cells cultured on 1, 20, 40, and 280kPa substrates, respectively. Error bars indicate standard deviation for BMDMs harvested from three mice and * $p < 0.05$ when compared to indicated condition by two-tailed Mann-Whitney U test. Source data including exact p-values are provided as a Source Data file.

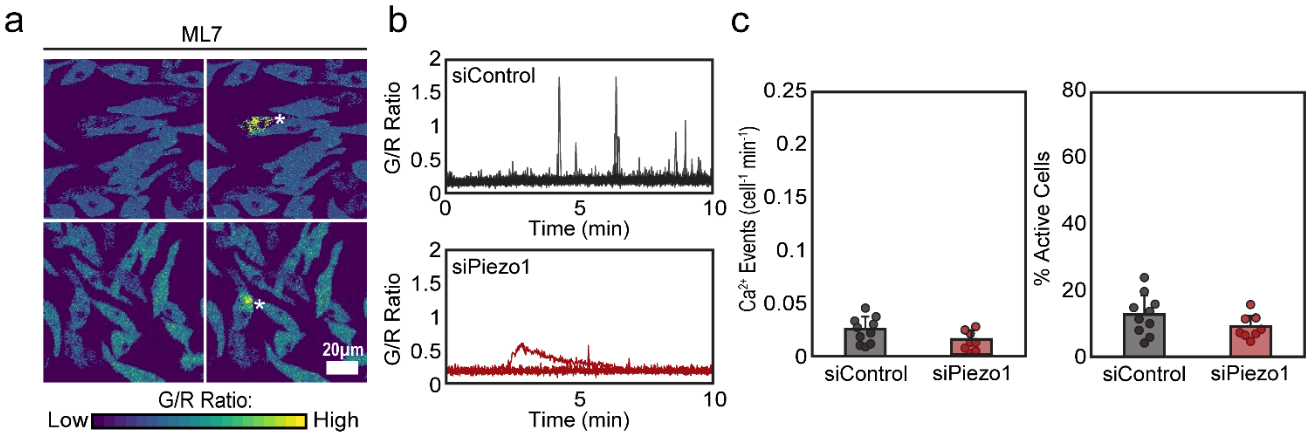

Supplementary Figure 14: Myosin light-chain kinase regulates Ca^{2+} influx in BMDMs. (a-c) Representative G/R ratio images of cell at rest (left) or (a), traces of individual Ca^{2+} events (b), and quantification of number of Ca^{2+} events (normalized for cell number and time, left) and fraction of cells showing Ca^{2+} elevations (right), (c) taken from a time-lapse video of siControl and siPiezo1 treated Salsa6f+ BMDMs following addition of DMSO or 25μM ML7 in Ringer solution containing 100ng/mL IFNγ/LPS. Asterisks denote the occurrence of a Ca^{2+} event. Each data point in (c) denotes a single video (N= 12 videos). Source data including exact p-values are provided as a Source Data file.
