# Supplementary material for: Mechanically activated ion channel Piezo1 modulates macrophage polarization and stiffness sensing
Source: Nat Commun. 2021 May 31;12:3256. doi: 10.1038/s41467-021-23482-5 (PMC8167181; doi:10.1038/s41467-021-23482-5)
Supplement: Supplementary file 3 — Description of Additional Supplementary Files [file 41467_2021_23482_MOESM3_ESM.docx]

**Description of Additional Supplementary Files**

**Supplementary Movie 1: Yoda1-induces Ca^2+^ influx in control but not siPiezo1 treated Salsa6f+ BMDMs.** BMDMs were treated with non-target (siControl) or Piezo1 (siPiezo1) siRNA and seeded on fibronectin coated MatTek dishes for 72 hours. BMDMs were first imaged at rest with Ringer solution followed by addition of 300nM Yoda1. Images were acquired using confocal microscopy and were processed in ImageJ to create a video of G/R ratio images over time.

**Supplementary Movie 2: IFNγ/LPS-induced Ca^2+^ influx in siControl and siPiezo1 treated Salsa6f+ BMDMs.** BMDMs were treated with non-target (siControl) or Piezo1 (siPiezo1) siRNA and seeded on fibronectin coated MatTek dishes for 72 hours. BMDMs were imaged following acute addition of Ringer solution (Unstim.) and Ringer solution containing 100 ng/mL IFNγ/LPS. Event location is indicated by asterisks and a still image summarizing event locations and numbers is included the end of the video. Images were acquired using confocal microscopy and were processed in ImageJ to create a video showing fluctuations in G intensity over time.

**Supplementary Movie 3: Millisecond scale Ca^2+^ events increase in frequency with macrophage activation.** Salsa6f+ BMDMs were seeded on fibronectin conjugated MatTek dishes and stimulated with media (Unstim.), IFNγ/LPS, and IL4/IL13 overnight prior to imaging. Images were acquired using TIRF microscopy and were processed by dividing each pixel by the mean pixel intensity to display F/F_0_ ratios over time.

**Supplementary Movie 4: IFNγ/LPS-induced Ca^2+^ influx in Salsa6f+ BMDMs cultured on different stiffness polyacrylamide gels.** BMDMs were seeded on fibronectin conjugated 1, 20, 40, and 280 kPa gels in MatTek dishes overnight. BMDMs were first imaged following acute addition of Ringer solution alone followed by Ringer solution containing IFNγ/LPS. Video provided is a max intensity projection of images taken over 3 different z-positions. Event location is indicated by asterisks and a still image summarizing event locations and numbers is included at the end of the video. Images were acquired using confocal microscopy and were processed in ImageJ to create a video showing fluctuations in G intensity over time.

**Supplementary Movie 5: Yoda1-induced Ca^2+^ influx in Salsa6f+ BMDMs cultured on different stiffness polyacrylamide gels.** BMDMs were seeded on fibronectin conjugated 1, 20, 40, and 280 kPa gels in MatTek dishes overnight. BMDMs were first imaged at rest with Ringer solution followed by Ringer solution containing 300nM Yoda1 and 5µM Yoda1. Video provided is a max intensity projection of images taken over 3 different z-positions. Images were acquired using confocal microscopy and were processed in ImageJ to create a video of G/R ratio images over time.
