# Supplementary material for: Mechanically activated ion channel Piezo1 modulates macrophage polarization and stiffness sensing
Source: Nat Commun. 2021 May 31;12:3256. doi: 10.1038/s41467-021-23482-5 (PMC8167181; doi:10.1038/s41467-021-23482-5)
Supplement: Supplementary file 9 — Reporting Summary [file 41467_2021_23482_MOESM9_ESM.pdf]

nature research

Corresponding author(s): Wendy Liu

Last updated by author(s): Mar 24, 2021

Reporting Summary

Nature Research wishes to improve the reproducibility of the work that we publish. This form provides structure for consistency and transparency in reporting. For further information on Nature Research policies, see our Editorial Policies and the Editorial Policy Checklist.

Statistics

For all statistical analyses, confirm that the following items are present in the figure legend, table legend, main text, or Methods section.

n/a	Confirmed	
☐	☒	The exact sample size (*n*) for each experimental group/condition, given as a discrete number and unit of measurement
☐	☒	A statement on whether measurements were taken from distinct samples or whether the same sample was measured repeatedly
☐	☒	The statistical test(s) used AND whether they are one- or two-sided<br>*Only common tests should be described solely by name; describe more complex techniques in the Methods section.*
☒	☐	A description of all covariates tested
☒	☐	A description of any assumptions or corrections, such as tests of normality and adjustment for multiple comparisons
☐	☒	A full description of the statistical parameters including central tendency (e.g. means) or other basic estimates (e.g. regression coefficient) AND variation (e.g. standard deviation) or associated estimates of uncertainty (e.g. confidence intervals)
☐	☒	For null hypothesis testing, the test statistic (e.g. *F*, *t*, *r*) with confidence intervals, effect sizes, degrees of freedom and *P* value noted<br>*Give P values as exact values whenever suitable.*
☒	☐	For Bayesian analysis, information on the choice of priors and Markov chain Monte Carlo settings
☒	☐	For hierarchical and complex designs, identification of the appropriate level for tests and full reporting of outcomes
☒	☐	Estimates of effect sizes (e.g. Cohen's *d*, Pearson's *r*), indicating how they were calculated

Our web collection on statistics for biologists contains articles on many of the points above.

Software and code

Policy information about availability of computer code

Data collection	Zen 2012 Black Edition, Nikon NIS-Elements, and Olympus Fluoview FV31S-SW Version 2.4.1.198 software were used for image acquisition.
Data analysis	ImageJ v1.8.0, MATLAB v2019b, Flika

For manuscripts utilizing custom algorithms or software that are central to the research but not yet described in published literature, software must be made available to editors and reviewers. We strongly encourage code deposition in a community repository (e.g. GitHub). See the Nature Research guidelines for submitting code & software for further information.

Data

Policy information about availability of data

All manuscripts must include a data availability statement. This statement should provide the following information, where applicable:

- Accession codes, unique identifiers, or web links for publicly available datasets
- A list of figures that have associated raw data
- A description of any restrictions on data availability

All data needed to evaluate conclusions are present in the paper and/or the Supplementary Information. Source data are provided with this paper. Additional data related to this paper may be requested from the authors.

Field-specific reporting

Life sciences study design

All studies must disclose on these points even when the disclosure is negative.

Sample size	Sample sizes were based on prior work and shown to generate reproducible results in independent experiments (Meli et al., Science Advances, 2020). Sample sizes are indicated in figure legends with n ≥ 5 used for in-vivo experiments and n ≥ 3 biological replicates used for in-vitro experiments.
Data exclusions	For live cell imaging, data points that included cell death (observed as extreme outliers) were removed. A total of one data point was removed for live cell imaging.
Replication	Replicates for each experiment were indicated in figure legends, all studies have been repeated at least twice with similar results.
Randomization	Animals were matched by age as well as gender and were exposed to the same treatment conditions (all animals were implanted with both soft and stiff materials), no randomization based on treatment groups was required.
Blinding	Blinding to genotype was used for both in vitro and in vivo while performing experiments. For in vitro experiments, analysis was performed without blinding to ensure correct comparisons are made within a Western blot, for example. For in vivo experiments, processing and analysis of tissues were performed with blinding.

Reporting for specific materials, systems and methods

We require information from authors about some types of materials, experimental systems and methods used in many studies. Here, indicate whether each material, system or method listed is relevant to your study. If you are not sure if a list item applies to your research, read the appropriate section before selecting a response.

Materials & experimental systems

n/a	Involved in the study	
☐	☒	Antibodies
☒	☐	Eukaryotic cell lines
☒	☐	Palaeontology and archaeology
☐	☒	Animals and other organisms
☒	☐	Human research participants
☒	☐	Clinical data
☒	☐	Dual use research of concern

Methods

n/a	Involved in the study	
☒	☐	ChIP-seq
☒	☐	Flow cytometry
☒	☐	MRI-based neuroimaging

Antibodies

Antibodies used	Antibody, Dilution (Application), Vendor (Cat. #)<br>iNOS, 1:1000 (WB), 1:100 (IHC), Abcam (ab15323)<br>ARG1, 1:1000 (WB), 1:50 (IHC), Abcam (ab60176)<br>GAPDH, 1:2000 (WB), BioLegend (607902)<br>PIEZO1, 1:1000 (WB), ProteinTech (15939-1-AP)<br>RFP, 1:1000 (WB), 1:400 (IF), Rockland (600-401-379)<br>NFκB, 1:1000 (WB), 1:800 (IHC), Cell Signaling (8242S)<br>p- NFκB, 1:1000 (WB), Cell Signaling (3033S)<br>NFκB, 1:100 (IF), Santa Cruz Biotechnologies (sc-8008)<br>STAT6, 1:1000 (WB), Cell Signaling (5397S)<br>p-STAT6 ,1:1000 (WB), Cell Signaling (56554S)<br>STAT1, 1:1000 (WB), Cell Signaling (14994)<br>p-STAT1, 1:1000 (WB), Cell Signaling (9167)<br>STAT3, 1:1000 (WB), Cell Signaling (9139)<br>p-STAT3, 1:1000 (WB), Cell Signaling (9145)<br>F4/80 ,1:200 (IHC), Fisher Scientific (50-112-9624)
Validation	All antibodies were purchased from the vendors noted above. All antibodies have been validated for the given applications in mouse, no further validation was performed.

Animals and other organisms

Policy information about studies involving animals; ARRIVE guidelines recommended for reporting animal research

Laboratory animals	Male and Female wild type, Piezo1fl/+, Piezo1ΔLysM, Salsa6f, and Piezo1P1-tdT mice were used to perform experiments.

Wild animals	This study did not use wild animals.
Field-collected samples	This study did not use field-collected samples.
Ethics oversight	University of California, Irvine Institutional Animal Care and Use Committee

Note that full information on the approval of the study protocol must also be provided in the manuscript.
